# The Biotechnological Application of Bacteriophages: What to Do and Where to Go in the Middle of the Post-Antibiotic Era

**DOI:** 10.3390/microorganisms11092311

**Published:** 2023-09-13

**Authors:** Su Jin Jo, Jun Kwon, Sang Guen Kim, Seung-Jun Lee

**Affiliations:** 1 College of Veterinary Medicine and Research Institute for Veterinary Science, Seoul National University, Seoul 08826, Republic of Korea; 2 Laboratory of Veterinary Public Health, College of Veterinary Medicine, Jeonbuk National University, 79 Gobong-ro, Iksan City 54596, Republic of Korea; 3 Department of Biological Sciences, Kyonggi University, Suwon 16227, Republic of Korea; 4 Department of Pharmaceutical Science and Engineering, Seowon University, 377-3 Musimseoro, Seowon-gu, Cheong-ju City 28674, Republic of Korea

**Keywords:** bacteriophage, phage therapy, phage engineering, phage display

## Abstract

Amid the escalating challenges of antibiotic resistance, bacterial infections have emerged as a global threat. Bacteriophages (phages), viral entities capable of selectively infecting bacteria, are gaining momentum as promising alternatives to traditional antibiotics. Their distinctive attributes, including host specificity, inherent self-amplification, and potential synergy with antibiotics, render them compelling candidates. Phage engineering, a burgeoning discipline, involves the strategic modification of bacteriophages to enhance their therapeutic potential and broaden their applications. The integration of CRISPR-Cas systems facilitates precise genetic modifications, enabling phages to serve as carriers of functional genes/proteins, thereby enhancing diagnostics, drug delivery, and therapy. Phage engineering holds promise in transforming precision medicine, addressing antibiotic resistance, and advancing diverse applications. Emphasizing the profound therapeutic potential of phages, this review underscores their pivotal role in combatting bacterial diseases and highlights their significance in the post-antibiotic era.

## 1. Introduction

Bacteriophages are viruses that selectively target and infect bacteria and offer various advantages over conventional antibiotics, including host-specific infections, self-amplification, coevolution, and adaptability [1,2]. Antibiotic resistance has recently emerged as a pressing global concern and presents a formidable public health challenge [3,4]. An alarming surge in bacterial resistance has rendered many once-effective antibiotics ineffective in combating bacterial infections [5]. Previously manageable infections, ranging from common urinary and respiratory tract infections to severe conditions such as sepsis and pneumonia, are becoming increasingly difficult to treat [6]. This has dire consequences for patient outcomes, exacerbating morbidity, mortality, and healthcare expenditure [7].

Adding to this complexity is the limited availability of novel antibiotics [8]. The development of novel antimicrobial agents has slowed owing to scientific, regulatory, and economic hurdles [9,10]. Compared with other therapeutic areas, pharmaceutical companies encounter difficulties recovering their investments in antibiotics research and development [11]. Consequently, the scarcity of new antibiotic compounds poses a challenge to antibiotic resistance. Immediate and collaborative actions are imperative to safeguard the efficacy of antibiotics and public health and to ensure the availability of effective treatments for future generations.

The search for viable alternatives to traditional antibiotics has become paramount because of the escalating threat of antibiotic resistance [12,13,14]. Bacteriophages, or phages, have emerged as promising candidates for addressing bacterial infections in the post-antibiotic era [15,16,17]. In addition to their therapeutic potential, bacteriophages have potential in the area of personalized medicine [18,19]. The isolation and tailoring of phages that target specific bacterial strains permit personalized treatment approaches. The next generation of phage therapies, involving genome engineering to customize phages according to the distinct characteristics of the infecting bacteria, could enhance their efficacy and contribute to more favorable patient outcomes [20,21]. Although using bacteriophages as antibiotic alternatives presents numerous advantages, challenges remain [22,23]. Issues related to phage production, quality control, safety, regulatory frameworks, and the potential for bacterial resistance necessitate ongoing research and development [24,25,26]. Addressing these challenges is pivotal for securing and effectively implementing bacteriophage therapy.

In this review, we highlight the recent advances in phage therapy as an alternative approach for treating bacterial diseases. Their specificity, self-replication, adaptability, synergy with antibiotics, and the potential for personalized medicine make them appealing options in the battle against antibiotic-resistant infections. Although further research and regulatory frameworks are imperative, we will explore the potential of bacteriophages as valuable tools in the post-antibiotic era.

## 2. Laboratory and Therapeutic Practices

### 2.1. Isolation and Characterization of Bacteriophages: Foundations for Therapeutic Applications

Isolation and characterization of bacteriophages are pivotal steps in laboratory research and therapeutic endeavors. These processes provide indispensable insights into the host specificity, morphology, genetics, life cycle, and antibacterial properties of phages [27,28,29]. This knowledge serves as a cornerstone for researchers and medical practitioners to understand the potential of phages in combating bacterial infections and tailor their application to precise and efficacious therapeutic interventions [30].

In both the laboratory and therapeutic contexts, the scrupulous isolation and thorough characterization of phage strains serve as the fundamental groundwork for comprehending their attributes and prospective roles in addressing bacterial infections. To date, numerous phages have been isolated, each targeting prominent bacterial pathogens, such as *Acinetobacter*, *Aeromonas*, *Erwinia*, *Mycobacterium*, *Pantoea*, *Pseudomonas*, *Salmonella*, *Staphylococcus*, *Streptococcus*, *Vibrio*, and *Xanthomonas* [31,32,33,34,35,36,37,38,39,40,41]. These phages span diverse sectors, including agriculture, food safety, veterinary practice, and human medicine.

Lytic phages, also referred to as virulent phages, are a category of bacteriophages that follow an obligatory lytic life cycle when infecting bacterial cells [42]. Within this cycle, the phage undergoes rapid replication within the host bacterium, ultimately culminating in the lysis (rupture) of the bacterial cell and the subsequent liberation of newly formed phage particles. This distinctive behavior has propelled lytic phages to the forefront of phage therapy [43,44,45].

In contrast, lysogenic phages exhibit an alternative pattern, refraining from lysing target bacteria [44]. Instead, they integrate genetic material into the bacterial genome to establish dormancy within the host [45]. Consequently, lysogenic phages have historically been excluded from therapeutic applications. Recent progress has revealed novel aspects of lysogenic phages and their potential therapeutic applications [46,47].

Once undervalued, lysogenic phages have garnered renewed attention owing to their unique characteristics and potential benefits in the therapeutic context [48,49]. Their capacity to facilitate controlled phage release, mitigate the risk of resistance development, enable gene transfer, and offer possibilities for gene therapy presents a compelling rationale for exploring lysogenic phages as valuable tools in the battle against bacterial infections and antibiotic resistance [50,51,52]. To fully utilize their therapeutic potential, further research is imperative to elucidate their mechanisms of action.

The potential of lytic and lysogenic phages as substitutes for antibiotics in therapeutic applications is widely acknowledged [53,54]. Although many studies postulate the therapeutic effect of phages, assessing the lytic potency of phages can be challenging, leading researchers to employ rapid, but sometimes incomplete, methods for its evaluation. The mathematical evaluation of lytic efficacy, such as the phagescore and virulence index, provides a precise measure of lytic activity, facilitating effective comparisons between different bacteriophages for specific applications and purposes [55,56]. This method aids in making informed decisions regarding the selection and utilization of bacteriophages in various research and therapeutic contexts. The use of such methods extends to various aspects of phage research, including phage screening, assessing different phage strains, mutants, infection conditions, host susceptibility, and even the formulation of phage cocktails [57,58].

These combined features offer a promising approach to addressing bacterial infections and antibiotic resistance [59,60]. By strategically utilizing the unique strengths inherent in both phage types, scientists and medical practitioners can formulate adaptable and efficacious therapeutic approaches that capitalize on their distinct advantages. This strategic integration is key to devising potent phage-based strategies to address the urgent challenges posed by the post-antibiotic era [13,44,61]. An in-depth investigation and study of their mechanisms and interactions is imperative to realize the complete therapeutic potential of lytic and lysogenic phages as indispensable alternatives to antibiotics.

### 2.2. Phage Therapy in Plant Agriculture

The use of antibiotics in plant agriculture has generated concerns regarding the potential emergence of antibiotic-resistant bacteria in the environment [62,63]. This raises concerns about the possible horizontal gene transfer of antibiotic-resistant genes from plant-associated bacteria to bacteria affecting human health [64,65]. Stringent regulations have been implemented to mitigate unnecessary and excessive antibiotic use in agriculture, permitting only a limited number of antibiotics to be used [66,67]. Consequently, alternative strategies such as phage therapy are garnering attention as prospective approaches for managing bacterial pathogens without exacerbating the antibiotic resistance crisis.

Within the framework of the One Health approach and the imposed restrictions on antibiotic overuse, the concept of harnessing naturally occurring phages from the environment has emerged as a viable strategy to promote sustainable agricultural practices [68,69]. In horticulture, phages exhibit considerable potential as a promising avenue to effectively manage bacterial outbreaks caused by pathogenic genera, such as *Agrobacterium*, *Dickeya*, *Erwnia*, *Pectobacterium*, *Ralstonia*, *Xanthomonas*, and *Xylella*, without the need for the overuse of antibiotics [28,41,70,71,72,73]. This approach is bolstered by the availability of commercially accessible phages that specifically target these pathogens, substantiating the viability of such strategies worldwide.

Ranjani et al. assessed the bacteriophage φXOF4 against *Xanthomonas oryzae*, the cause of bacterial leaf blight (BLB) in rice [74]. The efficacy of φXOF4 was observed across a wide range of hosts, effectively eradicating all pathogenic strains. Treatment with φXOF4 at a concentration of 1 × 10^8^ PFU/mL reduced the incidence of BLB and halted bacterial proliferation. Even at lower phage concentrations (1 × 10^7^, 1 × 10^6^, and 1 × 10^5^ PFU/mL), the disease was effectively controlled, with disease outbreak rates of 3.6%, 6.3%, and 15%, respectively. This study not only highlighted the stability of φXOF4, but also its dynamic population growth. Over 7 days, the initial bacterial population expanded from 1.65 × 10^6^ to 1 × 10^9^ CFU/mL, whereas the phage population declined from 1 × 10^8^ to 3 × 10^5^ PFU/mL.

Phage cocktails have emerged as a highly recommended strategy for optimizing the therapeutic potential of phages as biocontrol agents [75,76]. Phage cocktails encompass a combination of multiple phages targeting diverse bacterial strains or species, resulting in a broader spectrum of actions [77,78]. This approach enhances the probability of successfully eradicating bacterial populations, particularly when a single phage may prove insufficient owing to bacterial diversity or potential resistance. Carstens et al. isolated and characterized 29 phages that are virulent to *Pectobacterium atrosepticum*, a plant pathogenic bacterium that causes blackleg disease and potato soft rot [79]. Six phages were chosen to construct a phage cocktail based on their efficient propagation and genomic diversity, showing a remarkable host range of 93% against various tested *P. atrosepticum* strains. The resulting phage cocktail demonstrated significant efficacy in reducing disease incidence and potato soft rot severity by 61% and 64%, respectively, even under simulated storage conditions.

Several strategies involve the integration of antibiotics with phage cocktails to optimize the biocontrol potential of phages [80,81,82]. Kim et al. investigated the synergistic efficacy of a phage cocktail combined with kasugamycin, an aminoglycoside-class antibiotic used in agriculture for fire blight control [83]. Notably, the individual phages within the cocktail led to a reduction in *E. amylovora* cell count, ranging from 1.2 to 3.5 log CFU/mL, and a remarkable synergy among the phage cocktail was observed, resulting in a reduction of −3.7 log CFU/mL in vitro. Furthermore, the combined administration of a phage cocktail and antibiotics (minimum inhibitory concentration; MIC, 1/2MIC, and 1/4MIC) exerted a greater bactericidal effect than antibiotic-only treatment, indicating that antibiotic usage could be lowered to only a quarter. Antibiotics target specific bacterial structures or processes, hindering bacterial growth, while phages infect and rupture bacterial cells [84]. Combining antibiotics and phages creates a potent defense, challenging bacterial resistance on multiple fronts. This synergy is particularly effective against antibiotic-resistant strains, as phages can restore antibiotic effectiveness and enable lower antibiotic doses, reducing side effects and resistance development [85]. The combined action of antibiotics and phages, including phage-mediated weakening of bacterial cell walls, enhances their bactericidal effect, making this combination therapy a promising approach to combatting bacterial infections [86].

Numerous studies have explored the potential use of phages as biocontrol agents in the field [87,88,89]. Retamales et al. examined the application of bacteriophages to manage walnut blight caused by *Xanthomonas arboricola* pv. juglandis (*X. juglandis*) [90]. Three characterized phages, f20-Xaj, f29-Xaj, and f30-Xaj, showed specific lytic activity against *X. juglandis* strains from Chile and France. Notably, phage administration exhibited a dose-dependent protective effect in field trials. High doses of bacteriophages (4 cubic centimeters (cc)/L) demonstrated effectiveness comparable to that of Cu treatment in reducing leaf damage and disease incidence. The presence of bacteriophages in walnut tissues significantly decreased the bacterial load of *X. juglandis*, and high doses of bacteriophages (>3 cc/L) positively affected walnut fruit production. In a separate study by Rombouts et al., a six-phage cocktail was used to prevent bacterial blight caused by *Pseudomonas syringae* pv. *porri* [91]. This cocktail comprised five phages (vB_PsyM_KIL1-5) and one mutant phage (vB_PsyM_KIL3b) capable of infecting all 41 tested *P. syringae* pv. *porri* strains. Field trials showed variable results depending on the cultivar of plant and field, with disease protection (% disease incidence of phage treatment/% disease incidence of control) of 76–88% when infection was followed by phage treatment and 61–88% when the phage spray was followed by infection.

Similarly, the disease-preventive efficacy of phages has been validated, demonstrating comparability with the effects of antibiotics typically employed for each disease [92,93]. Several phages targeting *E. amylovora* resulted in an around 63% reduction in fire blight disease incidence, which is a comparable result with antibiotic (streptomycin) treatment in a 12-year-old Bartlett pear plant [92]. Recently, Nga et al. demonstrated the efficacy of three phage cocktails under greenhouse and field conditions [93]. The results revealed optimal disease control at phage concentrations of 10^7^ and 10^8^ PFU/mL. In field trials, both individual Φ31 phage and the phage cocktail effectively mitigated disease symptoms, reaching an efficacy comparable to the chemical bactericide oxolinic acid while significantly enhancing crop yield.

Prominent considerations in practical studies have underscored the significance of achieving persistence in the plant phyllosphere [94,95]. Research has been directed towards understanding the longevity of phages, encompassing repeated administration of phage solutions, devising strategies to shield phages from environmental stressors, and exploring in situ phage propagation through non-pathogenic phage-propagating (carrier) strains [96,97]. Born et al. researched formulations capable of protecting phages from ultraviolet (UV) radiation, a major environmental stress factor [95]. Various substances commonly found in the environment, such as beetroot and carrot juice, bovine milk casein, soy peptone, astaxanthin, amino acids, and Tween 80, yield positive outcomes by enhancing the half-life of phages against UV irradiation. Jo et al. developed a formulation incorporating Tween 80 and kaolin to shield phages from UV exposure, consequently bolstering their stability against UV radiation and promoting their adhesion to plant leaves to extend their persistence (Jo and Kim et al., in preparation). The concept of augmenting phage persistence through the use of carrier strains in the phyllosphere holds the potential for significant enhancement. Yet, the potential impact of carrier strains on disease incidence necessitates careful evaluation [97].

### 2.3. Phage Therapy in Veterinary and Human Medicine

The One Health approach acknowledges the interrelatedness of human, animal, and environmental health, underscoring their interdependence and close correlation [98]. Within this framework, the One Health perspective underscores the notable advantages of phages as precision-targeting agents to combat bacterial infections [99]. In contrast to antibiotics, which can exert a broad-spectrum effect on both detrimental and beneficial bacteria, phages exhibit an exceptional degree of specificity [100]. Each phage has evolved to recognize and infect distinct bacterial strains or species while preserving non-targeted bacteria. This precision is of paramount importance for maintaining the equilibrium of natural microbiota across humans, animals, and the environment [101,102].

Veterinary antibiotics have been used extensively in the livestock, poultry, and aquaculture industries for growth-promotion purposes [103,104]. The regulation of antibiotic use plays a pivotal role in curtailing potential misuse, which is a critical concern within the framework of One Health principles that encompass the comprehensive impact of antibiotic usage on human, animal, and environmental health [105]. It is crucial to understand that the widespread use of antibiotics promotes the spread of antibiotic resistance, underscoring the urgency of seeking alternative solutions [106]. Adherence to these criteria has led to a heightened interest in exploring alternative approaches for disease control, encompassing strategies such as vaccination, pre-/probiotics, and herbal extracts [107,108,109].

Phages have emerged as prominent candidates owing to their adaptable utility in therapeutic applications and vaccine development [110,111,112]. Numerous effective phages targeting major antibiotic-resistant bacterial pathogens, such as *Campylobacter*, *E. coli*, *Salmonella*, *Pseudomonas*, *Klebsiella*, *Acinetobacter*, *Staphylococcus*, and *Vibrio*, have been documented [113,114,115,116,117,118,119,120,121,122,123,124,125,126,127,128,129]. In this section, we selected articles that exemplify the accomplishments of phage therapy and its strategic implementation.

The increasing demand for organically grown fruits and vegetables underscores the necessity for safe soil amendments and organic fertilizers [130]. Nevertheless, they may contain harmful bacteria. Spreading them on the soil may contribute to the transfer of these microorganisms into the environment. Despite growing awareness regarding the potential hazards of pathogen contamination in crops, multiple foodborne diseases have been linked to fresh produce outbreaks [131,132]. To mitigate this risk, effective biocontrol methods employing bacteriophages were employed by several researchers. Grygorcewicz et al. demonstrated the potency of lytic *S. Enteritidis* phage, sall_v01, in swine manure, revealing a 99.6% and 99.98% reduction in bacterial count in the short (6 h) and long (14 h) term, respectively [133]. Spencer et al. showed the sanitizing effect against *S. Typhimurium* using a five-phage cocktail in dairy manure compost, observing a 99% and 99.9% reduction in bacterial count in the short (4 h) and long (34 h) term, respectively [134]. Moreover, the sanitizing application of antibiotics in food products is widely discouraged because of their extended environmental persistence and broad-spectrum antimicrobial effects. Bacteriophages provide precise and eco-friendly solutions for improving food safety [135]. Huang et al. demonstrated the potential of *Salmonella* phage LPSE1 to decontaminate in various ready-to-eat foods such as milk, sausage, and lettuce [136]. Bacteriophage ECPS-6 was excellent in removing *E. coli* O157:H7 in milk [137]. The phage ECPS-6 was able to reduce the *E. coli* O157:H7 both at room temperature (25 °C) and refrigerated condition (4 °C), leading to the reduction in the pathogen below the detection limit after 6 h with low phage concentration (5 × 10^6^ PFU/mL).

A notable investigation conducted by Hawkins et al. demonstrated the efficacy and safety of phage therapy in clinical trials involving companion animals, specifically dogs [119]. In this study, ten dogs afflicted with chronic *P. aeruginosa* otitis received a single 0.2 mL dose of six bacteriophages (BC-BP-01 to BC-BP-06; each of approximately 1 × 10^5^ PFU) administered directly into the external auditory canal using a sterile syringe. After 48 h, there was a significant 67% reduction in *P. aeruginosa* counts. Moreover, certain cases indicated the proliferation of multiple bacteriophage strains (5.9 × 10^7^ PFU/swab). Subsequently, an 18-month follow-up study was conducted, revealing sustained effectiveness, as evidenced by the consistent resolution of chronic ear infections and improvements in *P. aeruginosa* components that had been observed previously.

Notably, phage application may reduce antibiotic use by offering synergy through combination therapy [86,138,139]. By disrupting biofilm structures, phages enhance antibiotic penetration and increase bacterial susceptibility to their effects [140,141,142]. This collaborative effect holds promise for enhancing the treatment outcomes in challenging infections. Roszak et al. investigated bacteriophage–antibiotic combinations for combating biofilm by dual-species (*S. aureus* and Candida albicans) [143]. Phages and ciprofloxacin achieved a 90% reduction in mono-species biofilm-specific activity (BSA) and a 69% reduction in dual-species BSA, outperforming individual treatments. Kaźmierczak et al. revealed the potential of bacteriophages to outperform antibiotics, particularly against antibiotic-resistant *S. aureus* strains [144]. Three phages, vB_SauM-A, vB_SauM-C, and vB_SauM-D, showed effective antibiofilm eradication, reducing biofilm biomass and staphylococci count, which outperformed antibiotics both in vitro and in vivo (moth larvae) assay. Carvalho et al. observed the significance of developing formulations that shield phages from host immune defenses when administered orally [113]. Phages introduced through the gastrointestinal tract encounter various barriers, including exposure to gastric juice due to its potent acidity, which can lead to phage inactivation. Carvalho et al. further observed the absence of phages in fecal samples, indicating their susceptibility to low pH conditions. To counter this challenge, they employed antacids (30% CaCO_3_) combined with a phage solution, resulting in rapid bacterial inhibition starting 2 days after administration, leading to a substantial 30-fold reduction in campylobacteriosis incidence. Another study by Thanki et al. investigated the efficacy of phage therapy for mitigating *Salmonella* colonization in piglets using two phages, SPFM10 and SPFM14 [114]. Administering piglets an antacid solution (10% CaCO_3_) via oral gavage prior to daily feed introduction led to a significant reduction of 1.976 CFU/g in fecal *Salmonella* counts by day 3.

The immune system is another crucial factor impeding the therapeutic potential of phages [145,146,147]. Generally, phages are rapidly cleared (within a few days) from the circulatory system of animals [148,149,150]. Subsequent repeated administration for therapeutic efficacy may provoke immune stimulation, leading to counteractive phage clearance [151,152]. To address this challenge, Merril et al. employed natural selection to evolve phages with an extended circulation time [153]. Singla et al. employed liposome encapsulation to shield phages from neutralization through phage-specific antibodies, leading to an increased protection effect in vivo [154,155]. Kim et al. extended phage survival in vivo by encapsulating biocompatible substances, thereby mitigating the immune response to phages [156].

In advancing phage therapy, recent endeavors have been directed toward substantiating the safety and efficacy of phages through meticulous clinical trials [157,158,159]. The inaugural clinical trial, employing a phage cocktail identical to that used in the aforementioned canine otitis case, assumed the form of a Phase I/II assessment [157]. The primary objective of this trial was to evaluate the safety and efficacy of Biophage-PA at a low concentration (1 × 10^5^ PFU/mL). The trial included 24 patients with persistent ear infections caused by antibiotic-resistant *P. aeruginosa* strains. Over a comprehensive 42-day follow-up period, the findings showed a noteworthy reduction in *P. aeruginosa* counts within the phage-treated group (23.9%) when juxtaposed against the placebo cohort (108.9%).

Low titers of administered phages have emerged as a prominent factor contributing to the setbacks observed in clinical trials. A study conducted by Jault et al. exemplified this concern, as they aimed to address *P. aeruginosa* burn wound infections by utilizing 12 lytic phages (1 × 10^6^ PFU/mL) in conjunction with standard care for burn wound infections [158]. Regrettably, the trial was prematurely terminated owing to the inadequate efficacy of phage PP1131, which was potentially attributed to diminished phage concentrations during storage. Furthermore, Leitner et al. documented unfavorable outcomes in clinical trials involving male patients undergoing transurethral resection of the prostate [159]. This study aimed to evaluate the efficacy of intravesical bacteriophage therapy in managing urinary tract infections (UTIs). The success rates did not vary significantly among the phage, placebo, and antibiotic groups. These findings were influenced by several factors, including insufficient phage quantity in the pyophage group, which needs investigating to better understand hurdles for therapeutic success.

Conversely, a clinical investigation conducted by Ooi et al. adopted a higher phage concentration (3 × 10^9^ PFU), which yielded favorable outcomes and a notable absence of adverse effects within an experimental cohort subjected to an elevated phage dosage [160]. This observation indicates a positive therapeutic impact; nonetheless, meticulous evaluation during subsequent phase II clinical trials is imperative prior to establishing a comprehensive assessment.

Despite the promising therapeutic potential demonstrated in clinical trials, challenges related to phage stability in vitro and in vivo, immunogenicity, and efficacy remain unaddressed. Continued research endeavors, bolstered by the application of phage engineering, hold promise for enhancing therapeutic efficacy and stability [161]. This pursuit is vital for fully harnessing the formidable potential of phage therapy in the battle against infectious diseases, particularly for countering antibiotic-resistant superbacteria.

## 3. Biotechnological Approaches to Bacteriophage

### 3.1. Phage Engineering

Phage engineering, also known as phage modification or bioengineering, involves the manipulation of phages to amplify their therapeutic capabilities or broaden their utility [162,163]. This process employs diverse methods to alter phages, directing them towards specific bacterial strains, enhancing their stability during storage and transportation, bolstering their effectiveness, and facilitating the carriage of supplementary cargo, including therapeutic genes. Phage engineering has significant potential for customizing phage therapy, spanning applications from diagnostics to personalized medicine and bolstering the potency of phages against multidrug-resistant bacteria [164].

Phage display is a versatile and extensively employed engineering method that involves the genetic fusion of foreign peptides or proteins with the surface proteins of a bacteriophage [165]. This fusion empowers the phage to exhibit these peptides or proteins on its surface, enabling the selection of phages that specifically attach to designated targets such as bacterial surface components or host cells. This robust technique facilitates the screening and recognition of ligands or proteins with heightened affinity and specificity for desired targets, proving invaluable for drug discovery, diagnostics, and therapy [166,167,168]. However, challenges emerge owing to the intricate nature of the generated libraries, which could lead to gaps in target coverage [169]. The dimensions of the displayed peptides or protein domains are limited by the packaging constraints of the phage, thereby curbing the presentation of larger targets [170]. Furthermore, phage display requires labor-intensive and time-consuming processes for library construction and screening [171].

Recent advancements in the CRISPR-Cas system have ushered in a transformative era of bacteriophage engineering [172,173]. This system enables precise manipulation of phage genomes by utilizing guide RNA to direct the Cas enzyme to cut DNA at specific sites, allowing targeted genetic modifications, such as gene deletion, insertion, or alteration. This approach enhances phage properties, expanding lytic activity, host range, and the capacity to carry therapeutic cargo [174,175,176,177]. Moreover, it accommodates larger gene encoding because of increased genome packaging in comparison to smaller phages, such as M13, which are often used in phage displays. This progress has broadened the flexibility of phage displays and has supported the presentation of larger proteins or intricate gene constructs on phage surfaces [178]. Additionally, traditional labor-intensive screening involving radioactive isotopes or affinity tags for recombinant phages can be streamlined using guide RNA designed to target non-engineered (wild-type) phage-specific sequences [179].

Nonetheless, a cautious approach is imperative in phage engineering, considering the possible safety and regulatory implications. This approach ensures prudent and secure utilization of engineered phages in therapeutic and agricultural contexts. As ongoing research has expanded the boundaries of phage engineering, its capacity to transform disease management and infection mitigation continues to capture the attention of the biotechnology and medical industries.

### 3.2. Phages as Diagnostic Probes

Originally driven by the study of ligand–receptor interactions, phage display has evolved into a versatile platform encompassing a range of diagnostic methodologies. This technology has enabled the development of phage ELISA for antibody detection, biosensors for rapid pathogen identification, and peptide arrays for comprehensive biomarker screening [180]. The inherent signal amplification capacity of phages enhances their in situ detection and facilitates the identification of disease-specific bacterial markers. Moreover, phage-derived proteins hold promise for imaging and targeted drug delivery, thus advancing diagnostics and personalized medicine [181]. This adaptable tool fuels innovative strategies for disease detection and the exploration of biomarkers. Once engineered, these modified phages are useful in diverse diagnostic applications, offering sensitive and precise detection of disease-related entities in clinical samples, ranging from minute targets such as viruses to substantial entities such as cancer cells (Table 1) [182,183,184,185,186].

Anand et al. comprehensively outlined the application of phage displays for diagnosing coronaviruses [187]. Phage engineering has facilitated novel target exploration through epitope mapping, shedding light on interactions between coronaviruses, human cell receptors, and other molecules. Li et al. developed a method for detecting virus particles at levels lower than the minimum infective dose, such as 10^5^ copies/mL [188]. Similarly, Soendergaard et al. identified an optimal peptide for ovarian cancer diagnosis, enabling the direct application of radioactive indium (^111^In) labeling for conventional SPECT/CT instrumentation [189]. The use of M13 phages as detection probes provides an additional diagnostic avenue. Ferreira et al. employed an M13 phage display platform to visualize colorectal cancer cells via specific MCT1 marker binding [190]. Lee et al. encoded a lung-cancer-targeting peptide, Pep-1, on M13 phages, enabling non-invasive live in vivo imaging with a near-infrared microscope [191]. Salles et al. ingeniously mimicked a *Leishmania infantum* epitope using an M13 phage display, achieving 100% specificity and sensitivity for diagnosing human visceral leishmaniasis [192].

Phages possess a significant advantage in terms of their intrinsic host specificity, making them valuable tools for diagnosing and detecting specific bacterial hosts [193]. This attribute has been utilized to rapidly detect challenging-to-culture microorganisms such as *Mycobacterium* species [194,195]. Diverse approaches, including reporter proteins, phage amplification, and capture-based protocols, have been developed to facilitate the detection of pathogenic bacterial species [196,197].

Sarkis et al. devised recombinant bacteriophages by employing mycobacteriophage L5 to carry the firefly luciferase gene [194]. The limit of detection (LOD) was 70 CFU over 40 h. The use of temperate phages for reporter gene delivery has drawbacks, with infectivity potentially being restricted by superinfection exclusion and a limited host range, affecting platform versatility [193]. Riska et al. engineered the broad-host-range lytic phage TM-4 for luciferase delivery [195]. However, host cell lysis diminished the detectable light signal, reducing the sensitivity by 1000-fold. Conversely, Tanji et al. succeeded in rapidly detecting *E. coli*, both culturable and viable but non-culturable (VBNC), using lytic reporter phages [198]. Mutating phage-mediated lysis enabled prompt *E. coli* detection using the engineered phage T4e^−^/GFP, which discerned bacterial cells in both states within an hour. Developing a phage-amplification-based diagnostic protocol holds promise for harnessing structural GFP signals on phage surfaces.

Phage tail fibers can be used as diagnostic tools to harness the potent binding affinity of phages to bacterial surfaces. Denyes et al. devised a sensitive diagnostic assay for rapid *Salmonella* detection by utilizing a long-tail fiber (LTF) from bacteriophage S16 [199]. By conjugating it with horseradish peroxidase, they established an enzyme-linked LTF assay that achieved a sensitivity limit of detection (LOD) of 10^2^ CFU·mL^−1^. Similarly, Filik et al. exploited the tail fiber protein (TFP) of phage φYeO3-12 to detect *Yersinia enterocolitica*, which has a long incubation period (up to 10 days) [200]. They successfully engineered a maltose-binding protein-tagged TFP and employed an ELISA-based method, which yielded an LOD of 10^5^ CFU.

Similarly, available studies have highlighted that the sensitivity and specificity of phage-based methods exceed those of traditional approaches such as antibody-based diagnosis. Moreover, the cost and time advantages of phage industrialization are similar to those of eukaryotic cell-based antibody production. Exploring the potential of utilizing the complete phage structure as a detection tool augmented through phage genome engineering presents a promising route for maximizing innate host specificity.

**Table 1:** Application of phages for detection or diagnosis.

Category	Target	Platform	Method	Time	
Phage display	SARS-CoV-2	M13 phage for specific binding peptide screening	Fluorescent immunosensors	2 min	[188]
	Foot-and-Mouth Disease virus	M13 phage for specific binding peptide screening	ELISA	4 h	[182]
	Dengue 3 and 4 viruses	M13 phage	ELISA, Immunofluorescence assay	2 h	[183]
	Avian Influenza virus subtype H7N2	M13 phage	ELISA	6H	[184]
	*S. enterica* Enteritidis	M13 phage	Lateral Flow Assay	15 min	[185]
	*E. coli*, *S. aureus*, *P. aeruginosa*,	M13 phage	Raman spectroscopy	6 h	[186]
	*L. infantum*	M13 phage	ELISA	20 h	[192]
	Ovarian Cancer	M13 phage for specific binding peptide screening	SPECT/CT	N/A	[189]
	Colorectal Cancer	M13 phage for specific binding peptide screening	Imaging	N/A	[190]
	Lung Cancer	T7 phage	Protein Chip	3.5 h	[191]
Reporter Phage	*M. smegmatis*	Luciferase engineered mycobacteriophage L5	Luminescence assay	40 h	[194]
	*M. tuberculosis*	Luciferase engineered mycobacteriophage TM-4	Luminescence assay	Several minutes	[195]
	*E. coli*	GFP engineered phage T4	Fluorescent Microscopy	~1 h	[198]
Phage Tail Protein	*S. enterica*	Salmonell phage S16 long tail fiber protein	ELISA	2 h	[200]
	*Y. enterocolitica* serotype O:3	Yersinia phage φYeO3-12 tail fiber protein Gp17	ELISA	3 h	[200]

### 3.3. Phages as Carriers for Effective Genes/Proteins

Phage engineering confers phages with the ability to host and express functional proteins, thus increasing their status as versatile biotechnological instruments [174]. Customizing phages with specific proteins makes them flexible and robust agents that are well-equipped to tackle multifaceted challenges across biomedical and biotechnological domains. The inherent ability of phages to deliver DNA to bacterial hosts is a foundational trait that underscores their adaptability and efficacy in biotechnological applications. Based on this mechanism, several studies have pioneered innovative strategies for therapeutic intervention (Table 2).

Edgar et al. introduced an exemplary genetic approach utilizing temperate phage lambda to reinstate the drug sensitivity of drug-resistant pathogens residing on hospital surfaces [201]. The researchers have effectively sensitized drug-resistant *E. coli* strains to streptomycin and nalidixic acid by integrating engineered phage genomes carrying the wild-type genes (*rpsL* or *gyrA*, respectively). This intervention led to a significant decrease in the MIC of the respective antibiotics. The study highlights the potential of phages as delivery vectors to augment the efficiency of gene complementation and enhance the overall efficacy of the system.

A fascinating approach involves the utilization of modified phages carrying a natural bacterial defense mechanism, the CRISPR-Cas module, which can act as a “countermeasure” by specifically targeting antibiotic resistance genes [202]. Yosef et al. integrated the CRISPR-Cas system into the genome of the temperate phage lambda to specifically target antibiotic resistance genes, particularly ß-lactamases, such as *ndm* and *ctx*. The engineered CRISPR-Cas system demonstrated the ability to effectively identify and eliminate antibiotic-resistant plasmids in bacterial populations. This innovative strategy not only linked antibiotic sensitization and defense against lytic phages, but also showcased targeted prevention of horizontal gene transfer. By incorporating protospacers into lytic phages that match the target sites of the transferred CRISPR-Cas system, Yosef et al. achieved dual targeting of antibiotic-resistant genes and phages. This approach resulted in protection against specific lytic phages, selective hindrance of plasmid transformation, and the subsequent loss of antibiotic-resistant plasmids within phage-infected bacteria.

In a simplified manner, the investigation undertaken by Selle et al. involved the manipulation of *Clostridioides difficile* phage ФCD24-2 [203]. Specifically, this bacteriophage was subjected to genetic engineering, resulting in the incorporation of a spacer sequence with the precise intent of targeting a pivotal virulence factor regulator, RNase Y. This strategic modification induced irreparable damage to the bacterial genome, which was facilitated by the utilization of the host’s intrinsic CRISPR-Cas system. Concurrently, the strategic excision of the lysogeny module of the phage amplifies its therapeutic efficacy. However, careful modification when handling the lysogenic module is needed. This augmentation led to the demise of bacterial cells through a dual-faceted approach: first, the CRISPR-Cas system, with its precise targeting of the bacterial chromosome, induced irreversible genomic impairment; second, the orchestrated expression of phage holin and endolysin culminated in cellular lysis. The potential of this development to effectively target multiple genes poses a substantial and formidable challenge for bacterial pathogens.

Following phage infiltration of the host cell, a seizure of the host cell machinery transpires, instigating the replication of the phage’s genetic material and the assembly of fresh phage particles [204]. Concurrently, the transcriptional machinery of the host cells is inhibited. To demonstrate an effective approach, Lu et al. orchestrated engineering endeavors to leverage this phenomenon [205]. Through the conveyance of *lexA3* via the filamentous phage M13 (ФlexA3), they orchestrated an increase in the expression of the SOS response repressor in *E. coli*. This strategic intervention led to the pronounced suppression of the SOS network within *E. coli*. This orchestrated modulation notably intensified the effectiveness of quinolone antibiotics in both controlled laboratory settings and living organisms, ultimately yielding augmented survival rates in infected mice. Impressively, ФlexA3 showcased remarkable capabilities for augmenting antibiotic potency across diverse antibiotics, including quinolones, aminoglycosides, and β-lactams. Beyond their primary role in enhancing antibiotic activity, the engineered phages fulfilled a supplementary function as robust adjuvants, enhancing the antibiotic-induced eradication of bacteria while concurrently diminishing the presence of antibiotic-resistant persister cells. Additionally, their efficacy against biofilm-associated bacteria was notably improved. Lu et al. further investigated the targeting of diverse gene networks, including, but not limited to, s*oxR*, c*srA*, and *ompF*, with engineered phages, resulting in heightened antibiotic sensitivity and reduced biofilm formation.

Pei et al. meticulously aimed to tackle the intricate issues stemming from bacterial growth within biofilms, which contribute to unyielding biofouling in industrial operations and the persistence of infections within clinical domains [206]. Their efforts were centered on the genetic engineering of a T7 bacteriophage, endowing it with the capability to express the quorum-quenching enzyme AiiA. This enzyme can degrade acyl homoserine lactones (AHLs), which serve as critical components in bacterial intercellular communication, termed quorum sensing, a pivotal mechanism for biofilm formation. The tailored T7aiiA phage proved remarkably effective for AHL degradation across a spectrum of bacteria, concurrently inhibiting biofilm formation in mixed-species biofilms containing *P. aeruginosa* and *E. coli*. By lysing host bacteria and deploying quorum-quenching enzymes, these phages have demonstrated a versatile and all-encompassing strategy for confronting diverse bacterial populations within biofilm communities. This innovative avenue has significant potential for countering the multifaceted challenges associated with biofilm-related issues across various scenarios and environments.

Another study by Lu et al. conducted an innovative investigation that harnessed the synergistic potential of potent proteins and phage-mediated lysis for biofilm removal [207]. Their approach involved genetic modification of the lytic phage T7 to express the biofilm-degrading enzyme DspB during infection. This strategic maneuver yielded a remarkable reduction of approximately 99.997% in the bacterial cell count within the biofilm state, demonstrating an efficacy enhancement of nearly two orders of magnitude compared with non-enzymatic phage treatments. This significant improvement can be attributed to the successful integration of the DspB enzyme into the T7 phage, allowing for the precise and targeted eradication of biofilms. The study proposed the concept of generating libraries of enzymatically active phages to complement ongoing initiatives in searching for novel biofilm-degrading bacteriophages within the environmental milieu.

Lastly, cutting-edge platform technologies, such as SpyPhage, which involve the addition of tags to capsid proteins, offer the potential to further broaden the range of adaptable applications [208].

**Table 2:** Engineered bacteriophages carrying effector proteins.

Phage	Lifecycle	Target Bacteria	Carrying Gene	Potency	
Lambda	Temperate	*E. coli*	Antibiotic susceptible wild-type gene (rpsL for streptomycin/gyrA for nalixidic acid)	Sensitize the antibiotics by complementing the antibiotic susceptible wild-type gene	[201]
Lambda	Temperate	*E. coli*	CRISPR-associated genes (*cas3*, *cse1*, *cse2*, *cas7*, *cas5*, and *cas6e*) and spacer targetting ß-lactamases (*ndm* and *ctx*)	Sensitize the antibiotics by destroying antibiotic resistance conferring plasmids	[202]
ФCD24-2	Temperate	*C. difficile*	Spacer sequence targeting bacterial chromosome	Dual-faceted potency: CRISPR-Cas induced irreversible genomic impairment and phage lysis module mediated cellular lysis	[203]
M13	Chronic	*E. coli*	SOS response repressor, *lexA3*	Augmenting antibiotic potency by suppression of SOS response	[205]
T7	Lytic	*E. coli*	Quorum-quenching enzyme, AiiA	Biofilm degradation by degrading acyl homoserine lactones and bactericidal effect of inherent lytic potency of T7	[206]
T7	Lytic	*E. coli*	Biofilm detachment enzyme, DspB	Biofilm detachment by hydrolysis of N-acetyl-D-glucosamines found in the biofilm matrices using DspB and bactericidal effect of inherent lytic potency of T7	[207]
K1F	Lytic	*E. coli*	SpyTag	Provide versatility by tagging a variety of materials	[208]

## 4. Future Perspectives

The prospective landscape of phage therapy has immense potential, especially considering the strides made in phage engineering. This transformative approach empowers researchers to finely craft phages with enhanced attributes, including refined host specificity, bolstered stability, and amplified therapeutic potential. Especially, the production of engineered phages can be strengthened by the use of cell-free phage synthesis technology [209,210].

As envisioned by Pirnay, the trajectory of phage therapy points towards a realm of precision medicine akin to the current paradigm of cancer treatment [19]. This precision-engineered trajectory unveils avenues for the development of more potent and precisely targeted phage-based therapies, including the innovative realm of immunotherapy for combating bacterial infections, including those stemming from antibiotic-resistant strains. Moreover, the continuous deepening of our understanding of phage biology and genetic manipulation has led to further breakthroughs in phage therapy, making it a versatile and indispensable tool in the ongoing battle against infectious maladies.

In the field of phage research, a plethora of phages have been meticulously isolated and characterized, revealing noteworthy attributes, such as RNA-based genomic material, expansive genome sizes exceeding 200 kb, and the intriguing presence of a nucleus-like compartment safeguarding the phage genome from CRISPR nucleases [211,212,213]. Amidst this rich diversity, a noteworthy proportion of phage genes, termed the “dark matter”, or “ORFans” remain shrouded in mystery, their functions yet to be deciphered [214,215].

Determining these uncharted phage genes offers a captivating avenue for further exploration. Unveiling their roles holds promise for unearthing novel insights into phage biology and expanding the array of phage-driven tools for a myriad of applications, spanning disease diagnostics, therapeutics, and biotechnology. Continuous investigation, bolstered by phage engineering, is pivotal in order to fully harness their potential contributions to phage biology, the intricate interplay between phages, bacteria, and eukaryotic organisms, and a wide spectrum of biotechnological endeavors.

In summary, beyond the challenge of antibiotic-resistant superbacteria, a constellation of other intricate issues concerning bacteria, including immune evasion and the persistence of chronic infections via persister cells or VBNC states, are emerging as substantial concerns reminiscent of those faced in cancer research. In alignment with pioneering studies that have harnessed engineered phages as transformative breakthroughs, addressing these multifaceted conundrums requires innovative strategies. This pursuit encompasses the ongoing exploration of phage therapy as a versatile and potent tool in the ongoing quest to combat diverse bacterial threats.

## Data Availability

Not applicable.
